# Chronological changes in etiology, pathological and imaging findings in primary liver cancer from 2001 to 2020

**DOI:** 10.1093/jjco/hyae187

**Published:** 2025-01-07

**Authors:** Junya Tsuzaki, Akihisa Ueno, Yohei Masugi, Masashi Tamura, Seiichiro Yamazaki, Kosuke Matsuda, Yutaka Kurebayashi, Hiroto Sakai, Yoichi Yokoyama, Yuta Abe, Koki Hayashi, Yasushi Hasegawa, Hiroshi Yagi, Minoru Kitago, Masahiro Jinzaki, Michiie Sakamoto

**Affiliations:** Department of Radiology, Keio University School of Medicine, Tokyo, Japan; Division of Diagnostic Pathology, Keio University Hospital, Tokyo, Japan; Department of Pathology, Keio University School of Medicine, Tokyo, Japan; Department of Pathology, Keio University School of Medicine, Tokyo, Japan; Department of Pathology, Tokai University, School of Medicine, Kanagawa, Japan; Department of Radiology, Keio University School of Medicine, Tokyo, Japan; Department of Pathology, Keio University School of Medicine, Tokyo, Japan; Department of Pathology, Keio University School of Medicine, Tokyo, Japan; Department of Pathology, Brigham and Women's Hospital, Harvard Medical School, Massachusetts, USA; Department of Pathology, Keio University School of Medicine, Tokyo, Japan; Department of Radiology, Keio University School of Medicine, Tokyo, Japan; Department of Radiology, Keio University School of Medicine, Tokyo, Japan; Department of Surgery, Keio University School of Medicine, Tokyo, Japan; Department of Surgery, Keio University School of Medicine, Tokyo, Japan; Department of Surgery, Keio University School of Medicine, Tokyo, Japan; Department of Surgery, Keio University School of Medicine, Tokyo, Japan; Department of Surgery, Keio University School of Medicine, Tokyo, Japan; Department of Radiology, Keio University School of Medicine, Tokyo, Japan; Department of Pathology, Keio University School of Medicine, Tokyo, Japan; School of Medicine, International University of Health and Welfare, Chiba, Japan

**Keywords:** computed tomography, hepatocellular carcinoma, intrahepatic cholangiocarcinoma, primary liver cancer, subtype

## Abstract

**Purpose:**

To achieve a historical perspective, the chronological changes in primary liver cancer over a 20-year period were investigated at a single institution, focusing on shifts in etiology and the impact on imaging and pathological findings using The Liver Imaging Reporting and Data System.

**Materials and methods:**

A retrospective study of surgically resected primary liver cancer in 680 patients from 2001 to 2020 resulted in 434 patients with 482 nodules being analyzed. Dynamic contrast-enhanced computed tomography imaging and the Liver Imaging Reporting and Data System 2018 classification were employed. Two pathologists and two radiologists independently evaluated specimens and images.

**Results:**

This study highlighted a significant decline in cases of viral hepatitis and cirrhosis in primary liver cancer patients but an increase in intrahepatic cholangiocarcinoma and scirrhous hepatocellular carcinoma. Notably, there was a rise in non-viral hepatitis cases, potentially pointing toward an increase in steatohepatitic hepatocellular carcinoma cases in the future. Intrahepatic cholangiocarcinoma, scirrhous hepatocellular carcinoma and steatohepatitic hepatocellular carcinoma tumors exhibited slightly different distributions in the Liver Imaging Reporting and Data System classification compared with ordinary hepatocellular carcinoma, which may reflect the presence of fibrosis and lipid in tumor parenchyma.

**Conclusions:**

Consistent with past reports, this study demonstrated the emergence of primary liver cancer against a backdrop of non-viral and non-cirrhotic liver. Liver Imaging Reporting and Data System has been consistently useful in diagnosing primary liver cancer; however, among the histological subtypes of hepatocellular carcinoma, an increase is anticipated in scirrhous hepatocellular carcinoma and steatohepatitic hepatocellular carcinoma, which may present imaging findings different from those of ordinary hepatocellular carcinoma. This development may necessitate a reevaluation of the current approach for diagnosing and treating hepatocellular carcinoma based solely on imaging.

## Teaser

Primary liver cancer trends show an increase in non-viral, non-cirrhotic cases. Also, increments of intrahepatic cholangiocarcinoma and scirrhous hepatocellular carcinoma highlight a need to reassess diagnostic and management strategies.

## Introduction

Primary liver cancer (PLC), which includes hepatocellular carcinoma (HCC), combined hepatocellular-cholangiocarcinoma (cHCC-CC) and intrahepatic cholangiocarcinoma (ICC), is the sixth most common cancer and the third leading cause of cancer-related death worldwide [1].

HCC accounts for an estimated 75%–85% of all PLC cases and is frequently associated with virus-related liver cirrhosis (LC) [1,2]. In contrast to many other tumors that require histological diagnosis through biopsy or surgery for definitive diagnosis, for HCC, it has become standard in the clinical setting to proceed with diagnosis and treatment without obtaining histological findings, provided these typical findings, such as hyper-enhancement in the arterial phase and washout in the delayed phase, have been obtained in dynamic contrast-enhanced examinations [3]. The role of invasive biopsy has traditionally been relegated to PLC cases in which imaging results are ambiguous or prompt clinical debate, especially in cases of viral hepatitis. In recent years, however, a shift in the etiology of PLC has been observed. Numerous studies have reported a decline in viral hepatitis as a primary factor in the development of PLC. Concurrently, there has been a global rise in non-viral liver diseases, particularly metabolic dysfunction-associated fatty liver disease (MAFLD) and metabolic dysfunction-associated steatohepatitis (MASH) [4–8]. The introduction of direct-acting antivirals for the treatment of hepatitis C virus (HCV) has led to a decrease in HCV-related HCC cases, and in parallel, there has been an increase in the incidence of non-viral HCC [9,10]. In Japan, such trends are closely linked to the westernization of dietary habits, escalating obesity and lifestyle-related diseases, which are recognized as significant risk factors for liver cancer [11]. Furthermore, there is a growing trend in Japan of HCC developing in non-cirrhotic livers [12,13]. These evolving trends underscore a significant transformation in the landscape of liver disease etiology in Japan, where lifestyle factors are increasingly pertinent to liver health. This shift will likely have substantial implications for the imaging characteristics and pathological findings of PLC, highlighting the need for a comprehensive reevaluation of current diagnostic approaches and management strategies to consider these changing risk profiles and disease manifestations.

The Liver Imaging Reporting and Data System (LI-RADS), first established in 2011 and updated in 2018, has been instrumental in standardizing the radiological diagnosis of HCC [14]. This system offers a comprehensive framework for interpreting liver imaging findings, categorizing lesions to reflect the likelihood of HCC and guiding clinical management. LI-RADS is a highly capable tool for facilitating the diagnosis of HCC, particularly standing out for its specificity and positive predictive value [15]. However, LI-RADS is targeted at high-risk patients and those awaiting liver transplantation, and in the context of the changing patient backgrounds mentioned above, it is questionable whether traditional diagnostic methods should be directly applied. Moreover, to our knowledge, there have been no reports on this evolution of background liver conditions in terms of changes in radiological and pathological findings.

In the current study, we concentrate on the chronological developments observed in PLC over a 20-year period at our institution by scrutinizing the association between imaging features delineated by LI-RADS and patient outcomes. Our objective was to elucidate the influence of the shifting etiology of PLC on radiological and pathological investigations, thereby contributing to a deeper understanding of the interplay between evolving disease etiologies, imaging characteristics and patient prognosis in the context of liver malignancies. This approach is intended to refine diagnostic and prognostic strategies by aligning them more closely with the evolving landscape of PLC.

## Materials and methods

### Patients

We searched our institutional archival pathology database for a clinical cohort of PLC surveillance. We identified 680 patients who underwent surgical PLC resection between January 2001 and December 2020. Patients with a history of recurrent tumors or prior treatment for PLC were excluded to avoid confounding factors that could skew the imaging or histological findings interpretation. Dysplastic nodules and early HCC were excluded because these early lesions cannot be categorized into a subclass of HCC, and they lack early enhancement and are challenging to diagnose with dynamic computed tomography (CT). Carcinosarcoma and sarcomatous HCC were excluded because of the low number of cases. Tumors with an insufficient final diagnosis were also excluded. A total of 434 patients with 482 nodules were finally included in this study.

For imaging analysis, we utilized data collected after the introduction of the Picture Archiving and Communication System at our institution in 2006. We excluded 43 cases that did not include the late arterial phase in the dynamic CT scans and seven cases for which the tumors were not visible on the CT images. Furthermore, tumors showing cystic or non-mass forming patterns, such as intraductal papillary neoplasm of the bile duct and periductal infiltration-type ICC, were also excluded. After applying these criteria, we analyzed 233 patients with 258 nodules.

Also, we conducted survival analysis on 377 patients with a single nodule for each tumor type and each histological subtype of HCC.

**Figure 1:**
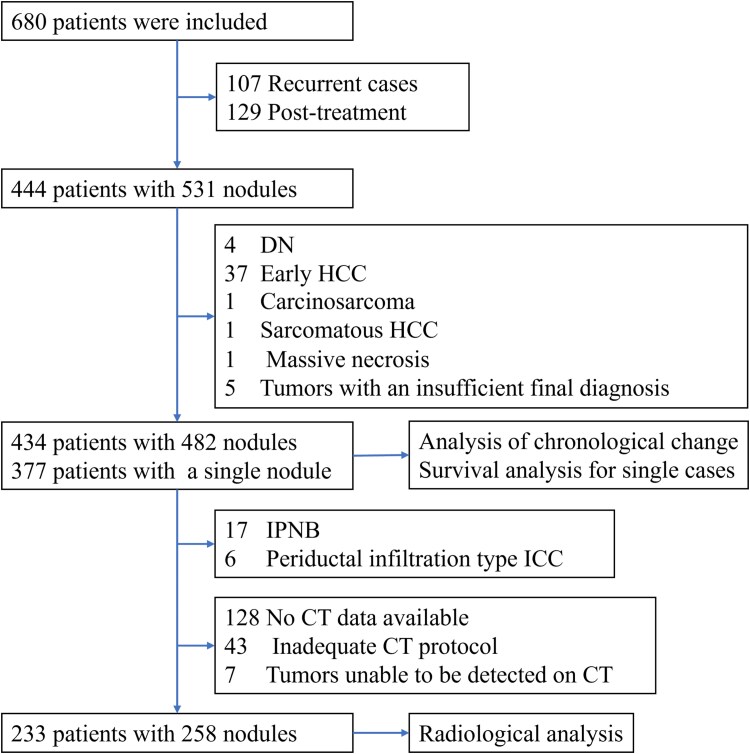
Flowchart of selection of the study population. DN, dysplastic nodule; HCC, hepatocellular carcinoma; IPNB, intraductal papillary neoplasm of the bile duct; ICC, intrahepatic cholangiocarcinoma; CT, computed tomography.

Comprehensive clinical data were recorded for each case, including age, sex, tumor markers, liver disease history and prognosis. The selection process and criteria are visually summarized in Fig. 1.

### Histopathological analysis

Two pathologists (U.A. and Y.M.) reviewed all histologic slides in consensus. The histological types of PLC and subtypes of HCC were classified according to the fifth edition of the WHO Classification of Digestive System Tumors of 2019 (Supplementary Fig. 1) [16–18]. Histologic features such as size and tumor differentiation grade were evaluated according to the classification proposed by the Liver Cancer Study Group of Japan [19], and the background liver fibrosis stage was assessed according to the METAVIR staging system [20].

### CT examination

CT studies were performed with 16- to 320-slice multidetector CT systems (LightSpeed16, LightSpeed VCT, Discovery CT 750 HD, Revolution; GE Healthcare, Waukesha, WI, USA; or Aquillion, Aquillion CX, or Aquillion ONE; Canon Medical Systems, Otawara, Japan). The dynamic CT protocol was as follows: initially, pre-contrast images of the liver were acquired, and each patient then rapidly received 600 mgI/kg of nonionic iodinated contrast agent using a power injector (DualShot GX, Nemoto Kyorindo, Tokyo, Japan) over 30 s. The first set of images was captured 40 s after starting the injection of the contrast agent and corresponded to the late arterial phase. Subsequent imaging was performed at 70–80 s to coincide with the portal venous phase and finally at 180 s to capture the delayed phase.

### Imaging analysis

Two radiologists (J.T and M.T), who were blinded to the results of the histopathological analysis, independently evaluated all images and classified them according to LI-RADS version 2018 definitions (Supplementary Fig. 2) [14]. We first classified lesions forming venous tumor thrombus as LI-RADS tumor in vein (TIV) and then classified lesions with a targetoid appearance as LI-RADS M. After that, we classified other tumors into LI-RADS categories 3–5 according to the presence or absence of arterial phase hyperenhancement, tumor size and major features such as enhancing capsule, non-peripheral washout and threshold growth if previous CT data were available. The classification was determined after consensus was achieved by discussion in cases where the original assessments differed.

### Statistical analysis

Cases were assigned to one of four 5-year intervals. Fisher’s exact test, Chi-square test, or analysis of variance was used to analyze chronological changes in etiology, liver fibrosis stage, patient background, PLC type, histological subtype of HCC and LI-RADS classification. As a post-hoc test, residual analysis was performed to investigate differences in the distribution of HCC histological subtypes based on the presence or absence of viral infection. Survival analysis was conducted using Kaplan–Meier curves and log-rank tests to assess overall survival (OS) and disease-free survival (DFS). Statistical significance was set at a *P*-value of < 0.05. All statistical analyses were performed using IBM SPSS Statistics, version 29 (IBM Corp., Chicago, IL, USA).

### Ethics approval

This study employed a retrospective design and was conducted following the principles of the 2013 Declaration of Helsinki and the 2018 Declaration of Istanbul. It was approved by the Ethics Committees of the Keio University School of Medicine (Shinjuku-ku, Tokyo, Japan, institutional review board no., 20 040 034).

## Results

### Characteristics of patients and tumors

In our analysis of 434 patients, 345 patients (79%) had HCC, 11 patients (3%) had cHCC-CC, 71 patients (16%) had ICC, four patients (1%) had both HCC and cHCC-CC and three patients (1%) had both HCC and ICC. Notably, viral hepatitis and cirrhosis were significantly more prevalent in patients with HCC than in patients with other types of PLC (both *P* < 0.001) (Table 1).

**Table 1:** Baseline characteristics of 434 primary liver cancer (PLC) patients

Patient characteristic	HCC	cHCC-CC	ICC	HCC + cHCC-CC	HCC + ICC	Overall	*P* value
Number of patients	345 (79%)	11 (3%)	71 (16%)	4 (1%)	3 (1%)	434	
Age							0.756
Median	68	69	69	66.5	68	68	
IQR	60–74	65–75	60–76	59.25–75.25	–	60–75	
Sex							0.094
Male	282 (82%)	11 (100%)	53 (75%)	2 (50%)	3 (100%)	351 (81%)	
Female	63 (18%)	0 (0%)	18 (25%)	2 (50%)	0 (0%)	83 (19%)	
Etiology							<0.001
Non-viral	140 (41%)	7 (64%)	55 (77%)	1 (25%)	1 (33%)	204 (47%)	
HBV	84 (24%)	3 (27%)	9 (13%)	0 (0%)	1 (33%)	97 (22%)	
HCV	117 (34%)	1 (9%)	7 (10%)	3 (75%)	1 (33%)	129 (30%)	
HBV-HCV	4 (1%)	0 (0%)	0 (0%)	0 (0%)	0 (0%)	4 (1%)	
LC							<0.001
Non-LC	249 (72%)	11 (100%)	56 (79%)	3 (75%)	3 (100%)	322 (74%)	
LC	91 (26%)	0 (0%)	4 (6%)	1 (25%)	0 (0%)	96 (22%)	
NA	5 (1%)	0 (0%)	11 (15%)	0 (0%)	0 (0%)	16 (4%)	
Tumor markers							
AFP (ng/ml)							<0.001
>300	57 (17%)	3 (27%)	0 (0%)	1 (25%)	1 (33%)		
<300	281 (81%)	8 (73%)	56 (79%)	3 75%	2 (66%)		
NA	7 (2%)	0 (0%)	15 (21%)	0 (0%)	0 (0%)		
PIVKA-II (mAU/mL)							< 0.001
>400	107 (31%)	5 (45%)	2 (3%)	2 (50%)	1 (33%)		
<400	211 (61%)	6 (55%)	47 (66%)	2 (50%)	2 (66%)		
NA	27 (8%)	0 (0%)	22 (31%)	0 (0%)	0 (0%)		
CEA (ng/ml)							0.665
>5	46 (13%)	1 (9%)	20 (28%)	0 (0%)	0 (0%)		
<5	163 (47%)	5 (45%)	48 (68%)	1 (25%)	2 (33%)		
NA	136 (39%)	5 (45%)	3 (4%)	3 (75%)	2 (66%)		
CA19–9 (U/ml)							< 0.001
>37	32 (9%)	3 (27%)	34 (48%)	0 (0%)	0 (0%)		
<37	127 (37%)	1 (9%)	27 (38%)	0 (0%)	1 (33%)		
NA	186 (54%)	7 (64%)	10 (14%)	4 (100%)	2 (66%)		

For the number of patients and the overall data, the percentages are based on the total number of patients (n=434). All other percentages are based on the total number of tumors of that type.

PLC, primary liver cancer; HCC, hepatocellular carcinoma; cHCC-CC, combined hepatocellular-cholangiocarcinoma; ICC, intrahepatic cholangiocarcinoma; IQR, interquartile range; HBV, hepatitis B virus; HCV, hepatitis C virus; LC, liver cirrhosis; AFP, alpha fetoprotein; PIVKA-II, protein induced by vitamin K absence or antagonist-II; CEA, carcinoembryonic antigen; CA19–9, carbohydrate antigen 19–9; NA, not available.

In the analysis of 482 nodules, 388 nodules (80%) were HCC, 16 nodules (4%) were cHCC-CC and 78 nodules (16%) were ICC (Supplementary Table 1). Among all HCCs, 254 nodules (65%) were categorized as ordinary, 51 nodules (13%) were scirrhous, 50 nodules (13%) were steatohepatitic, 16 nodules (4%) were macrotrabecular-massive (MTM), 11 nodules (3%) were clear cell and six nodules (2%) were lymphocyte rich. There were no cases of chromophobe, fibrolamellar, or neutrophil-rich HCC. No difference in tumor diameter was observed among the histological types of PLC (*P* = 0.884). However, among the histological subtypes of HCC, MTM was larger (*P* < 0.001) (Table 2).

**Table 2:** Baseline characteristics of 388 hepatocellular carcinoma (HCC) nodules

HCC nodule characteristic	Ordinary	Scirrhous	SHCC	MTM	Clear cell	Lymphocyte rich	Overall	*P* value	
Number of nodules	254 (65%)	51 (13%)	50 (13%)	16 (4%)	11 (3%)	6 (2%)	388	
Size (mm)								<0.001
Median	32	25	23	74	25	23	30	
IQR	20–48.75	18–37	18–32.5	33.5–92.75	14–31	17–57.5	20–47.5	
Differentiation								<0.001
Well	47 (19%)	4 (8%)	8 (16%)	0 (0%)	1 (9%)	1 (17%)	61 (16%)	
Moderate	170 (67%)	40 (78%)	40 (80%)	0 (0%)	10 (91%)	1 (17%)	261 (67%)	
Poor	37 (15%)	7 (14%)	2 (4%)	16 (100%)	0 (0%)	4 (66%)	66 (17%)	
Etiology								0.003
Non-viral	98 (39%)	20 (39%)	30 (60%)	1 (6%)	3 (27%)	2 (33%)	154 (40%)	
Viral	156 (61%)	31 (61%)	20 (40%)	15 (94%)	8 (73%)	4 (67%)	234 (60%)	

For the number of nodules and the overall data, the percentages are based on the total number of HCC nodules (n=388). All other percentages are based on the total number of nodules of that subtype.

PLC, primary liver cancer, HCC, hepatocellular carcinoma, cHCC-CC, combined hepatocellular-cholangiocarcinoma; ICC, intrahepatic cholangiocarcinoma; SHCC, steatohepatitic hepatocellular carcinoma; MTM, macrotrabecular massive type; IQR, interquartile range.

**Figure 2:**
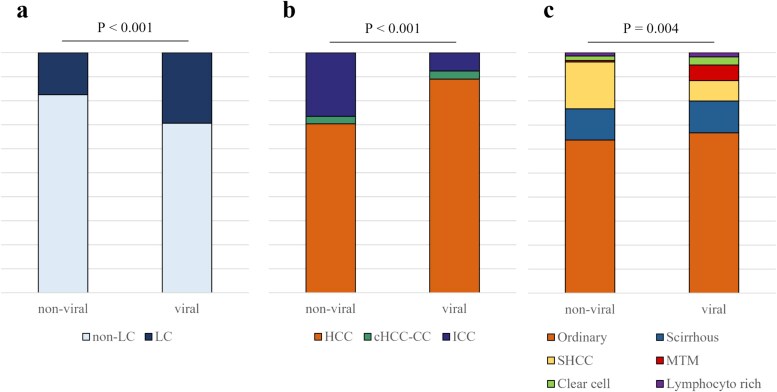
Breakdowns for non-viral patients and patients with viral hepatitis in terms of the proportion of primary liver cancers (PLCs) with liver cirrhosis (LC) (**a**), the histologic types of PLC (**b**) and the histologic subtypes of HCC (**c**). In PLC derived from non-viral patients, the proportion of cirrhosis was significantly lower, the proportion of HCC was significantly higher, and among the HCC subtypes, the proportion of SHCC was significantly higher. PLC, primary liver cancer; HCC, hepatocellular carcinoma; cHCC-CC, combined hepatocellular-cholangiocarcinoma; ICC, intrahepatic cholangiocarcinoma; SHCC, steatohepatitic; HCC, hepatocellular carcinoma; MTM, macrotrabecular-massive.

In the non-viral subset of patients, there was a significantly higher occurrence of non-cirrhotic liver compared with those with viral hepatitis origins (*P* < 0.001) (Fig. 2a). The non-viral group also exhibited a significantly lower proportion of HCC but a higher incidence of ICC (*P* < 0.001) (Fig. 2b). Among the subtypes of HCC, a significant difference in distribution was observed based on the presence or absence of viral infection (*P* = 0.004). Residual analysis revealed that steatohepatitic HCC (SHCC) was significantly more prevalent in non-viral livers (*P* = 0.003), while MTM was significantly more common in viral livers (*P* = 0.008). No significant differences in distribution were observed for other histological subtypes. (Fig. 2c).

### Imaging analysis

**Figure 3:**
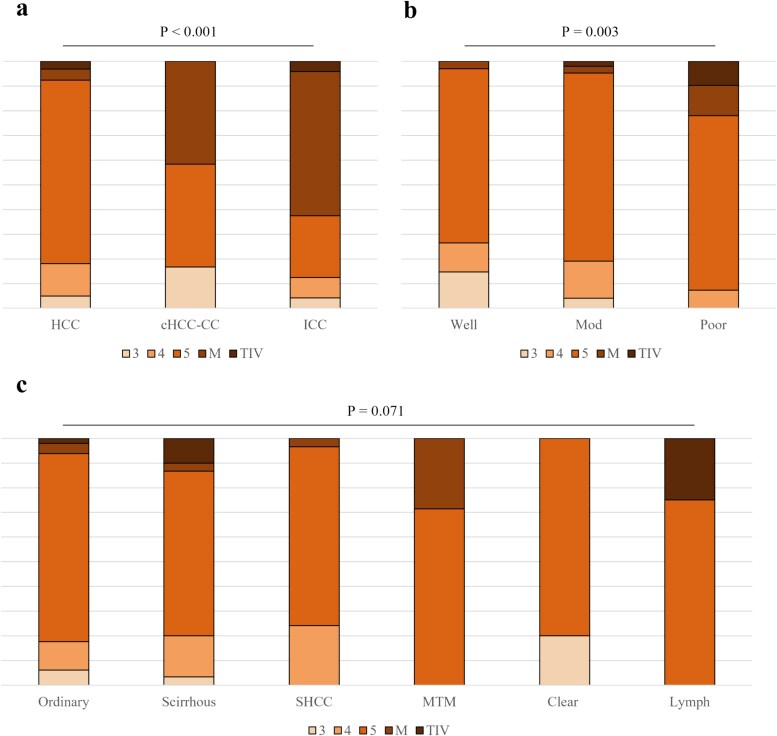
LI-RADS classification of histologic types of PLC (**a**), tumor differentiation of HCC (**b**) and histologic subtypes of HCC (**c**). The numbers in parentheses represent the number of nodules for each category. The LI-RADS classification varied significantly by histological type, with cHCC-CC and ICC tending to present as LI-RADS M. Additionally, among HCC, well-differentiated tumors tended to present as LI-RADS 3 or 4, while poorly differentiated tumors tended to present as LI-RADS M. No significant differences were observed among the histological subtypes of HCC. LI-RADS, the liver imaging reporting and data system; PLC, primary liver cancer; HCC, hepatocellular carcinoma; TIV, tumor in vein.

Among the histologic types of PLC, HCC showed a significantly higher frequency of being categorized as LI-RADS 5, whereas cHCC-CC and ICC more frequently presented as LI-RADS M (*P* < 0.001) (Fig. 3a). Among HCCs, there was a significant correlation between the LI-RADS classification and tumor differentiation (*P* = 0.003) (Fig. 3b). There was a slight difference in LI-RADS classification among the subtypes of HCC without statistical significance (*P* = 0.071) (Fig. 3c). Compared with ordinary HCC, scirrhous HCC showed a slightly higher frequency of LI-RADS 4 and TIV and SHCC showed a slightly higher frequency of LI-RADS 4.

### Analysis of chronological changes

**Figure 4:**
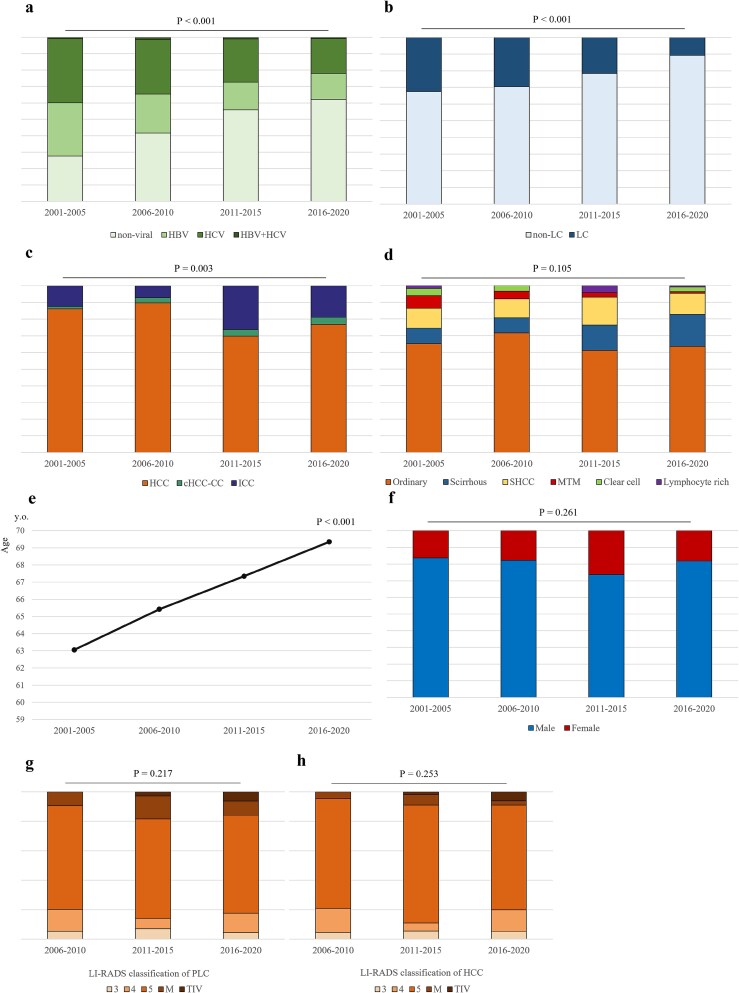
Chronological changes between 2001 and 2020 in the proportion of non-viral hepatitis (**a**), non-LC (**b**), histologic type of PLC (**c**), histologic subtype of HCC (**d**), mean age at onset of PLC (**e**) and sex ratio (**f**). Chronological changes in the breakdown of the LI-RADS classification of PLC (**g**) and HCC (**h**). Over time, PLC derived from non-viral and non-cirrhotic liver conditions showed an increasing trend, with ICC also trending upward in histological type. Although no significant changes were observed overall among the histological subtypes of HCC, scirrhous HCC exhibited a slightly increasing trend. Regarding patient background, the age of onset tended to increase, while the gender ratio remained unchanged. Additionally, no chronological changes were observed in the LI-RADS classification on imaging findings. LC, liver cirrhosis; PLC, primary liver cancer; HCC, hepatocellular carcinoma; cHCC-CC, combined hepatocellular-cholangiocarcinoma; ICC, intrahepatic cholangiocarcinoma; SHCC, steatohepatitic HCC; MTM, macrotrabecular-massive HCC.

Between 2001 and 2020, there were notable shifts in the causes of PLC. There were significant declines in the number of patients with PLC stemming from viral hepatitis and LC, with both changes being statistically significant (*P* < 0.001) (Fig. 4a and b).

Among PLC types, the proportion of ICC has been relatively high since 2011, demonstrating a statistically significant overall increasing trend (*P* = 0.003) (Fig. 4c). While no statistically significant changes were observed in HCC histological subtypes (*P* = 0.105), scirrhous HCC displayed a gradual upward trend. (Fig. 4d).

Additionally, the average age at which patients were diagnosed with PLC significantly increased (*P* < 0.001) (Fig. 4e). The distribution of cases by sex remained relatively stable over time, with no significant changes (*P* = 0.261) (Fig. 4f).

For imaging findings, LI-RADS 5 was the most frequent classification across all intervals, and no significant changes were observed overall for either PLC (*P* = 0.217) or HCC (*P* = 0.253) (Fig. 4g and h).

### Survival analysis

**Figure 5:**
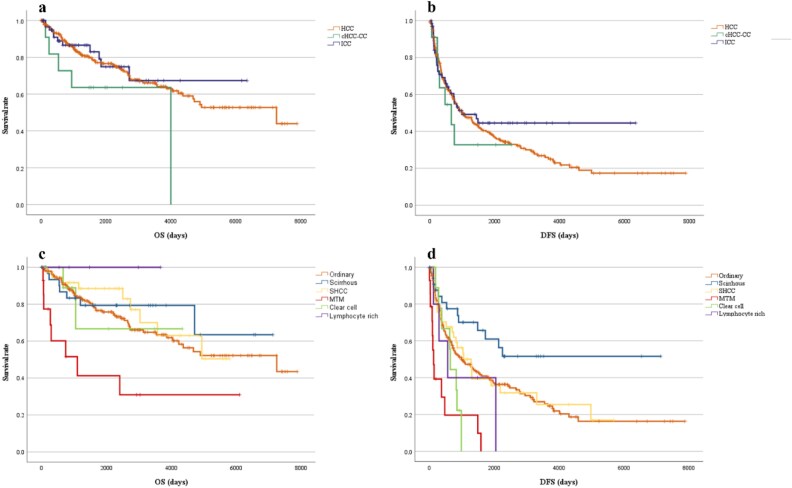
The OS (**a**) and DFS (**b**) of each PLC type and the OS (**c**) and DFS (**d**) of each HCC subtype. Each histological type exhibited similar OS and DFS. As for the subtype of HCC, scirrhous HCC demonstrated a longer DFS; however, this did not significantly impact OS. OS, overall survival; DFS, disease-free survival; PLC, primary liver cancer; HCC, hepatocellular carcinoma.

Each histological type exhibited similar OS and DFS. As for the subtype of HCC, scirrhous HCC demonstrated a longer DFS; however, this did not significantly impact OS. (Fig. 5).

## Discussion

Recent reports have indicated a decrease in the prevalence of viral hepatitis and an increase in liver damage due to lifestyle-related diseases [3–8]. In this context, we evaluated the chronological changes between 2001 and 2020 in PLC distribution among patients who underwent surgical resection at our institute. We found an increase in the proportion of non-viral and non-cirrhosis patients with PLC at our institute, which is consistent with the findings of previous studies [9–13]. Among PLC types, ICC showed a significant increasing trend. Among the HCC subtypes, scirrhous HCC exhibited a slight increasing trend, although no significant difference was observed overall, likely due to the low frequency of scirrhous HCC. While no distinct trends were observed in the other histological subtypes, the increasing incidence of cases arising from non-viral liver conditions suggests the potential for a future increase in SHCC. In Japan, a nationwide follow-up survey of PLC is conducted every 2 years by the Liver Cancer Study Group of Japan. According to their reports, of the HCCs surgically resected in 2000–1, 42.6% were associated with LC. However, this percentage had decreased to 34.4% by 2012–13, indicating an increasing trend in the development of HCC from non-cirrhotic liver, a trend that the current study showed to be ongoing. Furthermore, during the same period, the proportion of ICCs among surgically resected PLCs increased from 6.0% to 8.5%, indicating a trend similar to that observed in the current study [12,13].

MAFLD is the most common chronic liver disease in Western countries and has recently been recognized as a risk factor for the development of HCC; moreover, MAFLD can present with HCC without any underlying cirrhosis [21]. A large meta-analysis conducted by Stine et al. [22] reported that, in non-cirrhotic livers with MASH, the incidence of HCC was 2.7 times higher than that of HCC in non-cirrhotic livers resulting from other causes. SHCC was first reported by Salomao et al. [23] in 2010 and was newly added as a histological subtype of HCC in the 5th edition of the WHO classification in 2019 [16]. SHCC is reportedly associated with lifestyle-related diseases such as obesity and hypertension [24,25]. In our study also, the incidence of SHCC was higher in non-viral livers, suggesting that an increase in the occurrence of SHCC may be expected in the future, although this trend is not yet reflected in current data.

The proportions of the various LI-RADS classifications for PLC and HCC have remained relatively consistent between 2001 and 2020, underscoring its continued utility as a diagnostic methodology despite the evolving landscape of underlying liver pathologies.

The LI-RADS classification demonstrated substantial variability across histological types, with HCC most frequently classified as LI-RADS 5 and ICC as LI-RADS M. Given that LI-RADS is primarily designed to detect HCC, it appears to achieve this objective effectively. Although no statistically significant differences were observed among HCC histological subtypes overall, minor variations were noted: scirrhous HCC exhibited a slightly higher frequency of LI-RADS M classification, while SHCC was marginally more often classified as LI-RADS 4.

On pathological examination, scirrhous HCC and SHCC show intratumoral fibrous stroma [16,26,27]. This fibrosis impacts contrast enhancement, affecting dynamic CT scan enhancement patterns. Yoshikawa et al. [28] reported that regions with histological fibrosis exhibited delayed enhancement in contrast-enhanced CT. Maehara et al. [29] reported that tumors with histological fibrosis, specifically higher levels of intratumoral collagen, were associated with heterogeneous enhancement in the arterial phase and persistent enhancement in the equilibrium phase. This implies that, for these subtypes of HCC with fibrosis, there may be a reduced probability of meeting the major criteria of LI-RADS, such as arterial phase hyperenhancement and non-peripheral washout. Furthermore, Kim et al. [30] and Lee et al. [31] reported that scirrhous HCC shows atypical features on dynamic CT, such as peripheral rim-like enhancement in the arterial and portal phases, the presence of prolonged or delayed enhancement and the absence of delayed washout. In LI-RADS, tumors with rim-like enhancement in the late arterial phase are classified as having a targetoid appearance and categorized as LI-RADS M. Specifically regarding SHCC, Inui et al. [32] reported that 20% of SHCCs appear hypo-attenuating or isoattenuating in the arterial phase, suggesting that SHCCs tend to less frequently demonstrate the typical LI-RADS 5 appearance. Delagnes et al. [33] have reported that because fat-suppressed images are used in contrast-enhanced MRI, HCCs with a high-fat content inherently exhibit low signal intensity in pre-contrast images and are less frequently observed to demonstrate arterial phase hyperenhancement in the early phase compared with HCCs with less fat content. In LI-RADS, the presence of fat within a tumor is listed as an ancillary feature, suggesting HCC. However, upgrading from LI-RADS 4 to LI-RADS 5 based solely on ancillary features is not permitted. Similarly, in evaluating dynamic CT imaging, the presence of fat may also affect the LI-RADS classification. The LI-RADS primarily targets high-risk patients, especially those with virus-related LC. However, as revealed in the present study, the background of PLC is shifting towards an increased incidence of cases arising from non-viral and non-cirrhotic backgrounds. As mentioned above, tumors such as scirrhous HCC and SHCC, which may be less likely to exhibit typical LI-RADS 5 features, are either on the rise or potentially increasing. Consequently, relying solely on imaging-based diagnoses to determine treatment strategies, as is currently practiced, could lead to inadequate diagnostic and therapeutic outcomes. This possibility should be carefully considered.

Also, we evaluated the prognosis by tumor type and subtype of HCC. Our findings indicate that scirrhous HCC had a longer DFS, although this did not significantly influence OS. There remains no consensus on the prognosis of scirrhous HCC; for example, Huang et al. [34] reported poorer OS in Barcelona Clinic Liver Cancer (BCLC) stage B patients with scirrhous HCC, while prognosis was comparable in patients with BCLC stages 0 + A and C. Due to the rarity of scirrhous HCC, comprehensive prognostic studies remain limited, highlighting the need for further case accumulation. The observed extension of DFS suggests a reduced risk of recurrence, which may improve patients’ quality of life and hold clinical significance. Although OS was unaffected, prolonged recurrence control may enhance the potential to benefit from emerging therapies. Additionally, the lack of OS improvement in this study may reflect not only recurrence-related mortality but also the impact of underlying LC, underscoring the distinct prognostic value of DFS in patient management.

This study has several limitations. First, it was carried out at a single institution, which could introduce a certain level of bias in the selection of cases. This might have affected the representativeness of the sample compared with a broader, more diverse population. Second, this study exclusively targets cases treated with surgical resection. Advanced cancers with distant metastases and recurrent cases were excluded. However, given that the results of this study are based on rigorous pathological evaluation, there is a possibility that these findings could be applicable to similar cases. With the accumulation of further studies, the need for pathological evaluation before initiating treatment may also emerge for cases deemed inoperable. Third, the imaging findings of this study are based on consensus achieved between two radiologists. This approach might only partially capture the range of differences in interpretation that can occur in the classification of LI-RADS.

In conclusion, this study reveals a shift in liver cancer epidemiology, with fewer cases linked to viral hepatitis and cirrhosis and a rise in ICC and scirrhous HCC. While LI-RADS remains a reliable diagnostic tool, some histologic subtypes of HCC, particularly those with fibrosis, are less frequently classified as LI-RADS 5. This is due to the specific pathological traits of these subtypes and could influence future imaging diagnostics of liver malignancies. The current study highlights the necessity for continuous evolution and refinement in imaging criteria within LI-RADS to ensure its effectiveness and relevance in adapting to the changing landscape of liver cancers.

## Supplementary Material

Chronological_changes_in_PLC_JJCO_Supp_hyae187(1)
