# Supplementary material for: Chronological changes in etiology, pathological and imaging findings in primary liver cancer from 2001 to 2020
Source: Jpn J Clin Oncol. 2025 Jan 7;55(4):362–71. doi: 10.1093/jjco/hyae187 (PMC11973632; doi:10.1093/jjco/hyae187)
Supplement: Chronological_changes_in_PLC_JJCO_Supp_hyae187(1) [file chronological_changes_in_plc_jjco_supp_hyae187(1).docx]

**Chronological changes in etiology, pathological, and imaging findings in primary liver cancer from 2001 to 2020**

Authors:

Junya Tsuzaki 1), Akihisa Ueno 2,3)*, Yohei Masugi 3,4), Masashi Tamura 1), Seiichiro Yamazaki 3), Kosuke Matsuda 3,5), Yutaka Kurebayashi 3), Hiroto Sakai 1), Yoichi Yokoyama 1), Yuta Abe 6), Koki Hayashi 6), Yasushi Hasegawa 6), Hiroshi Yagi 6), Minoru Kitago 6) , Masahiro Jinzaki 1), Michiie Sakamoto 3,7)*

* Corrensponding Authors

1) Department of Radiology, Keio University School of Medicine, Tokyo, Japan.

2) Division of Diagnostic Pathology, Keio University Hospital, Tokyo, Japan.

3) Department of Pathology, Keio University School of Medicine, Tokyo, Japan.

4) Department of Pathology, Tokai University, School of Medicine, Kanagawa, Japan.

5) Department of Pathology, Brigham and Women's Hospital, Harvard Medical School, Massachusetts, USA.

6) Department of Surgery, Keio University School of Medicine, Tokyo, Japan.

7) School of Medicine, International University of Health and Welfare, Chiba, Japan.

Table of Contents

Supplementary table…………………….………………………………………………….…………1

Supplementary figures and figure legends……………………………………………………………2

**Supp. Table** Baseline characteristics of 482 PLC nodules from 434 patients

| PLC nodule characteristic | HCC | cHCC-CC | ICC | Overall | p-value |
| --- | --- | --- | --- | --- | --- |
| Number of nodules | 388 (80%) | 16 (3%) | 78 (16%) | 482 |  |
| Size (mm) |  |  |  |  | 0.884 |
| Median | 30 | 35 | 33.5 | 30 |  |
| IQR | 20–47.5 | 20–60 | 23–50 | 20–48 |  |

**Supplementary Figures and Figure Legends**

**
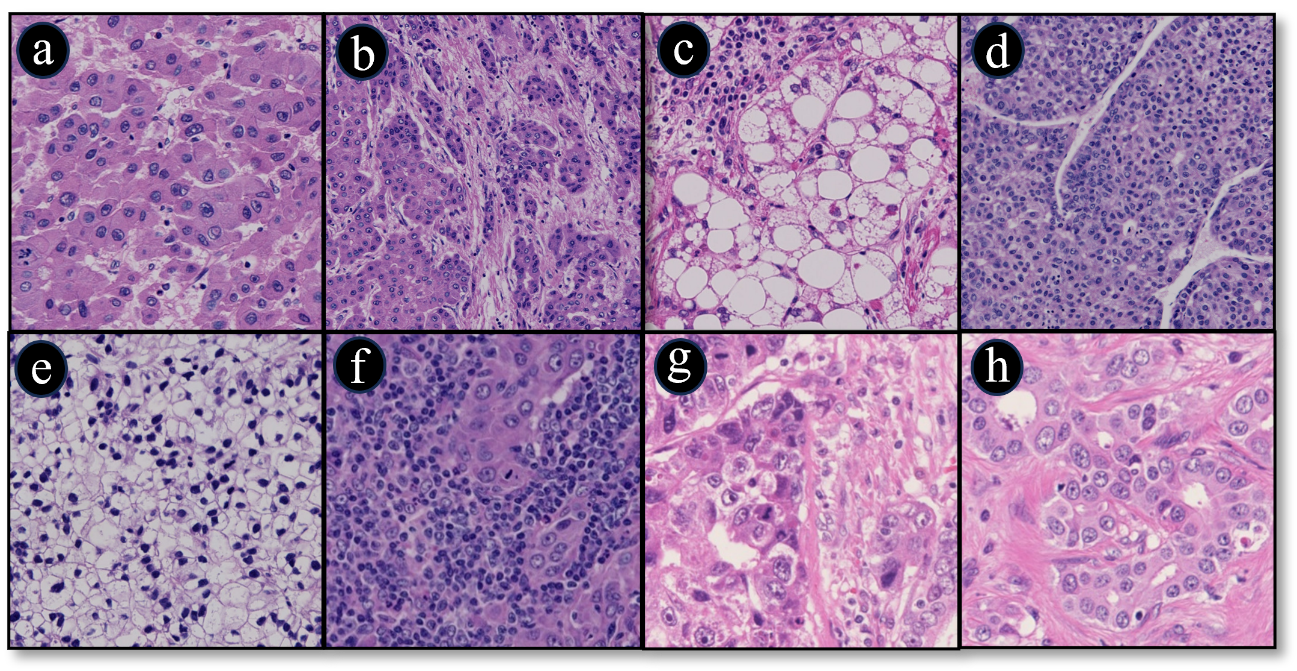
**

**Supp. Fig 1** Representative histologic images of each HCC subtype, cHCC-CC, and ICC. (**a**) Ordinary HCC, (**b**) scirrhous HCC, (**c**) steatohepatitic HCC, (**d**) macrotrabecular-massive HCC, (**e**) clear cell HCC, (**f**) lymphocyte rich HCC, (**g**) cHCC-CC, (**h**) ICC. (a) Ordinary HCC represents atypical hepatocytic proliferation with principal histological growth patterns, such as trabecular, solid, pseudoglandular, and macrotrabecular patterns, but without special subtype patterns. (b) Scirrhous HCC is defined as more than 50% of the tumors showing dense intratumoral fibrosis. (c) Steatohepatitic HCC shows tumor steatosis, fibrosis, and inflammation, such as steatohepatitis. (d) Macrotrabecular-massive HCC is defined as more than 50% of tumors showing a macrotrabecular pattern. (e) Clear cell HCC is defined as more than 80% of the tumors showing clear cell morphology from glycogen accumulation. (f) Lymphocyte rich HCC is defined as a tumor with numerous lymphocytic infiltrations, as lymphocytes outnumber tumor cells. (g) cHCC-CC is a tumor with both HCC and ICC components. (h) ICC is adenocarcinoma with biliary differentiation and shows a variable-sized ductal or tubular pattern. HCC = hepatocellular carcinoma, cHCC-CC = combined hepatocellular-cholangiocarcinoma, ICC = intrahepatic cholangiocarcinoma.


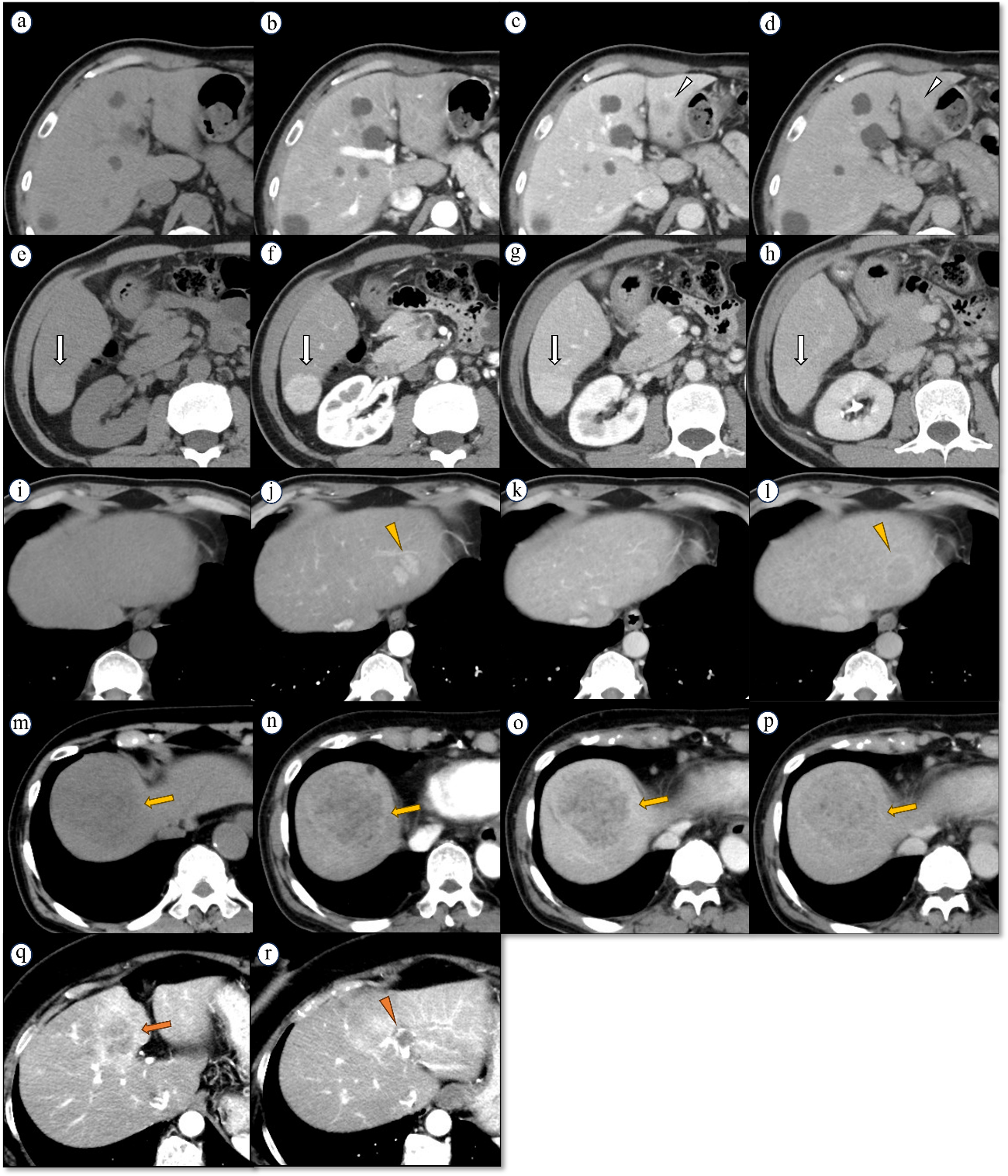


**Supp. Fig.** **2** Representative images for each LI-RADS classification.

The first row (**a-d**): A 17 mm HCC classified as LI-RADS 3 (white arrowhead). (**a**): pre-contrast, (**b**): LAP, (**c**): PVP, (**d**): EP. This tumor is small and does not exhibit early enhancement, but it shows washout in the PVP and the EP and was classified as LI-RADS 3.

The second row (**e-h**): A 21 mm steatohepatitic HCC classified as LI-RADS 4 (white arrow). (**e**): pre-contrast, (**f**): LAP, (**g**): PVP, **(h)**: EP. The tumor exhibits early enhancement, but there is no apparent washout in the EP or capsular enhancement, consequently, it is categorized as LI-RADS 4.

The third row (**i-l**): A 28 mm HCC classified as LI-RADS 5 (yellow arrowhead). (**i**): pre-contrast, (**j**): LAP, **(k)**: PVP, (**l**): EP. The tumor shows early enhancement, washout in the EP, and capsular enhancement, consequently regarded as LI-RADS 5.

The fourth row (**m**-**p**): A 68 mm scirrhous HCC classified as LI-RADS M (yellow arrow). (**m**): pre-contrast, (**n**): LAP, (**o**): PVP, (**p**): EP. The internal area of the tumor does not exhibit enhancement in the early phase but shows rim-like arterial enhancement. Therefore, this tumor is regarded as LI-RADS M.

The last row (**q**.**r**): A 30mm poorly differentiated HCC (**q**, orange arrowhead) accompanied by a tumor thrombus in the portal umbilical portion (**r**, orange arrow). A tumor thrombus continuous with the portal vein is observed. Therefore, this tumor is regarded as LI-RADS TIV.

LI-RADS = The Liver Imaging Reporting And Data System, HCC = hepatocellular carcinoma, LAP = late arterial phase, PVP = portal venous phase, EP = equilibrium phase
